# A Novel Murine Model of Inflammatory Bowel Disease and Inflammation-Associated Colon Cancer with Ulcerative Colitis-Like Features

**DOI:** 10.1371/journal.pone.0041797

**Published:** 2012-07-27

**Authors:** Laura P. Hale, Paula K. Greer

**Affiliations:** Department of Pathology, Duke University Medical Center, Durham, North Carolina, United States of America; Charité, Campus Benjamin Franklin, Germany

## Abstract

Mutations that increase susceptibility to inflammatory bowel disease (IBD) have been identified in a number of genes in both humans and mice, but the factors that govern how these mutations contribute to IBD pathogenesis and result in phenotypic presentation as ulcerative colitis (UC) or Crohn disease (CD) are not well understood. In this study, mice deficient in both TNF and IL-10 (T/I mice) were found to spontaneously develop severe colitis soon after weaning, without the need for exogenous triggers. Colitis in T/I mice had clinical and histologic features similar to human UC, including a markedly increased risk of developing inflammation-associated colon cancer. Importantly, development of spontaneous colitis in these mice was prevented by antibiotic treatment. Consistent with the known role of Th17-driven inflammation in response to bacteria, T/I mice had elevated serumTh17-type cytokines when they developed spontaneous colitis and after systemic bacterial challenge via NSAID-induced degradation of the mucosal barrier. Although TNF production has been widely considered to be be pathogenic in IBD, these data indicate that the ability to produce normal levels of TNF actually protects against the spontaneous development of colitis in response to intestinal colonization by bacteria. The T/I mouse model will be useful for developing new rationally-based therapies to prevent and/or treat IBD and inflammation-associated colon cancer and may further provide important insights into the pathogenesis of UC in humans.

## Introduction

Human inflammatory bowel disease (IBD) typically manifests as either ulcerative colitis (UC) or Crohn’s disease (CD). Patients with UC or CD both suffer from episodic bloody diarrhea and abdominal pain, but they differ in the gross and histologic distribution of their intestinal inflammation. The inflammation in UC always involves the rectum and may also extend proximally in a continuous fashion to involve the sigmoid, descending colon, or the entire colon (“pancolitis”). Histologically, crypt abcesses and ulceration are common in UC, with inflammation typically limited to the mucosa. In contrast, CD can involve any part of the gastrointestinal tract, although the most common disease patterns involve terminal ileum alone, colon alone, or both terminal ileum and colon. Inflammatory lesions in CD extend deep into the intestinal wall (“transmural”), may include non-caseating granulomas, and are characteristically separated by uninvolved tissue (“skip lesions”). Complications of transmural involvement in CD include perforations, formation of large abcesses, and abnormal connections (fistulas) between adjacent bowel loops or the body surface.

Mutations that increase susceptibility to IBD have been identified in a number of genes in both humans and mice. Some of these mutations are seen in human families with both UC and CD, but the factors that govern whether UC or CD occurs in any given patient have not been determined. This information is vitally important clinically, since these diseases have a different prognosis and respond differently to available therapies. For example, homozygous mutation in the receptor for IL-10 was recently found to be a cause of early onset, severe CD in humans [1]. However, genome-wide association and other studies have also identified polymorphisms in the human IL-10 gene that confer an increased risk for developing UC [2], [3]. The immunoregulatory cytokine IL-10 is important for generation and function of T regulatory cells that have been shown to protect against IBD development in murine models [4], [5]. IL-10 was also recently shown to decrease IL-1β production by dendritic cells, thus down-regulating the Th17-mediated inflammation that has been implicated in IBD pathogenesis [6].

Although mice with deletion of the gene that encodes IL-10 were originally reported to spontaneously develop colitis [7], we have found that *Il10* ^−/−^ mice on the C57BL/6 background are resistant to the development of spontaneous colitis when kept free of pathogens such as *Helicobacter* [8], [9]. However, these *Il10* ^−/−^ mice readily develop moderate to severe IBD when triggered by events that compromise their mucosal barrier, such as infection with Helicobacter species [8], [10] or exposure to non-steroidal anti-inflammatory drugs (NSAIDs) [11], [12]. IBD in *Il10* ^−/−^ mice is characterized by transmural inflammation and skip lesions and is typically most severe in the cecum and the proximal colon. We have also occasionally observed granulomas, entero-entero or entero-cutaneous fistulas, and abdominal abcesses in *Il10* ^−/−^ mice with long-standing intestinal disease (L.P. Hale, unpublished observations). *Il10* ^−/−^ mice thus appear to provide an IBD model that closely resembles what is seen in humans with CD.

The cytokine TNF is a major regulator of inflammation. In addition to its direct effects, TNF induces many gene products involved in the inflammatory pathway, tissue repair, and immune responses, including IL-1, IL-6, and prostaglandins. Consistent with its role in regulating inflammation, TNF-neutralizing monoclonal antibodies such as infliximab have been shown to significantly decrease inflammatory activity in treatment-resistant CD in humans, to enhance closing of fistulas, and to be an effective maintenance therapy in patients with either luminal or fistulizing CD [13]–[16]. Efficacy of infliximab has also been shown in humans with UC [17]. The clinical success of these specific anti-TNF therapeutics has led TNF to be regarded as a major regulator of inflammation in IBD. However, the lack of efficacy of other anti-TNF drugs such as the soluble TNF receptor etanercept [18] and the TNF synthesis inhibitor LMP-420 [19] in human or murine IBD has raised questions about whether anti-TNF therapies in IBD work via neutralization of TNF or via some other mechanism (e.g. immunosuppression generated by cytotoxicity to TNF-expressing cells). To further address the importance of TNF in the pathogenesis of IBD, we created *Il10* ^−/−^ mice that were also globally deficient in TNF and assessed their susceptibility to colits, inflammation-associated colon cancer, and systemic responses to enteric bacteria.

## Materials and Methods

### Animal Studies

Breeding pairs of C57BL/6 mice deficient in IL-10 (strain name  =  *B6.129P2-Il10^tm1Cgn^/J*; stock # 002251) and TNF (strain name  =  *B6.129S6-Tnf^tm1Gkl^/J*; stock # 005540) were obtained from Jackson Laboratories (Bar Harbor, ME) and crossed to generate *Tnf ^+/−^ Il10^−/−^* (T-het/I) and *Tnf ^−/−^ Il10^−/−^* double knockout (T/I) mice that were IBD-susceptible, with heterozygous or complete deficiency of TNF production in all cell types. The breeding scheme typically used generated T-het/I and T/I mice as littermates that would be exposed to the same maternal microbiota at birth. When possible, co-housed same-sex littermates of differing genotypes were used when age-matched comparisons were made. However, each genotype group studied was composed of multiple litters and the data obtained was not matched to littermates.

Mice were housed in polycarbonate micro-isolator cages in individually ventilated racks under specific pathogen-free barrier conditions, with access to food and water *ad libitum*. Sentinel mice exposed repetitively to dirty bedding from the mice used in this study were negative for parasites by microscopic exam, negative for *Citrobacter rodentium* by fecal culture, negative for infection with *Helicobacter* species by PCR of feces, and negative by serology for a panel of 22 murine protozoal, bacterial, and viral pathogens, including murine parvovirus, murine hepatitis virus, and murine norovirus.

For studies of spontaneous colitis, cohorts of mice were euthanized for histologic scoring of colon inflammation at pre-determined time points or if they reached the humane endpoints of rectal prolapse, loss of >15% body weight, or signs of pain and distress including poor grooming, decreased activity, and hunched posture. Some cohorts of mice were exposed to 200 ppm piroxicam in powdered LabDiet 5001 chow (Land O’Lakes-Purina, Richmond, IN) for 7 days, a treatment previously shown to trigger the onset of colitis in *Il10* ^−/−^ mice [11], [12]. Based on measured food consumption, the dose of piroxicam averaged 48 mg/kg/day in these experiments. The mice were then placed back on chow without piroxicam and observed for an additional 16 days before euthanasia for histologic scoring of colon inflammation.

For studies of colitis prevention, antibiotic therapy was initiated at the time of weaning using commercially available rodent chow that contained 3 mg amoxicillin, 0.5 mg clarithromycin, 1 mg metronidazole, and 20 µg omeprazole per 5 g wafer (Bio-Serv, Frenchtown, NJ). Control groups were given identical wafers that lacked antibiotics.

### Tissue Analysis

After euthanasia, the digestive tract was divided into 5 segments representing the cecum, and proximal, mid-, distal, and terminal colon/rectum. Tissues were fixed in Carnoy’s solution for 2–4 hrs or in 10% neutral buffered formalin for 18 hrs, then processed into paraffin blocks. The severity of colonic inflammation and incidence of colon neoplasia seen in hematoxylin and eosin-stained sections was scored by a board-certified pathologist blinded to experimental group. Histologic scores were calculated as described [12], [20], using a scale from 0–75 that takes into account mucosal changes in the 5 different bowel segments, including hyperplasia and ulceration, degree of inflammation, and % of each bowel segment affected by these changes. Using this scale, a score >12 indicates the presence of colitis, 13–24 indicates mild colitis, and <26 indicates moderate to severe colitis. Animals with histologic scores that fall into the moderate to severe range typically have either scattered severe inflammatory lesions or extensive disease involving the mucosa. Sections were also scored for non-invasive or invasive neoplasia [21]. Gastrointestinal intraepithelial neoplasia (synonymous with atypical hyperplasia, microadenoma, carcinoma *in situ*) and adenoma were considered to be non-invasive lesions. A diagnosis of invasive carcinoma required the presence of a desmoplastic response (formation of an abundant collagenous stroma) to differentiate invasion from mucosal herniation or pseudoinvasion. Regions of neoplasia that were separated by regions of normal mucosa were scored as separate lesions.

### Cytokine Responses to Bacteria and their Products

To assess systemic cytokine responses to bacterial products, adult mice (>8 wks) were injected intraperitoneally with 100 µg lipopolysaccharide (LPS) from *E. coli* O55:B5 in 1 ml of saline. Animals were euthanized 90 min later to obtain serum for cytokine analysis. Alternately, mice were placed on diets containing 200 ppm piroxicam and serum was obtained 42 hrs later. The cytokines present in serum were quantitated by comparison to standards of known concentrations using either enzyme immunoassays (Mouse TNFα DuoSet DY410; R & D Systems, Minneapolis, MN) or a Luminex bead-based fluorescent multiplex immunoassay (Millipore, Billerica, MA). The lower limit of detection was defined as the concentration that yielded a signal <2 SD above background; this varied from 1 to 10 pg/ml for the analytes tested. Measurements below this limit were assigned a value of zero. According to the manufacturer’s literature, the reagents used had negligible cross-reactivity with other analytes.

### Statistical Analysis

Statistical comparison of histologic scores and cytokine levels for the genotypes studied was performed using ANOVA, then a post-test for multiple comparisons using GraphPad Prism software, version 5.03. The cytokine data was log-transformed before statistical analysis. A Dunnett’s post-test was used when each group was compared against the wild type control. A Tukey’s post-test was used when experimental groups were compared against each other. Categorical data was compared via chi-squared analysis (Fisher’s exact test). Survival rates were calculated using Kaplan-Meier test with *p*-values calculated using the log rank test (MediCalc Software, Version 12.2.1, Mariakerke, Belgium). A p value ≤0.05 was considered to represent a significant difference between groups.

### Ethics Statement

All animal studies were approved (protocol number A151-09-05) by the Institutional Animal Care and Use Committee of Duke University, an institution accredited by the Association for Assessment and Accreditation of Laboratory Animal Care (AAALAC), International. Although colitis has the potential to produce pain and distress, analgesics were not employed in these studies, since non-steroidal anti-inflammatory agents (NSAIDs) can exacerbate colitis and opioids affect intestinal motility and function. Suffering was minimized by providing euthanasia for mice that met humane endpoints, as specified above.

## Results

### Effect of TNF Deficiency on Breeding Success and Growth of *Il10* ^−/−^ Mice

*Tnf* ^−/−^ *:Il10* ^−/−^ (T/I) young adult males and females were fertile with normal litter sizes, however pups often exhibited high early mortality and/or failure to thrive. The breeding life of T/I dams was typically limited to 1–2 litters and some never got pregnant at all. Pregnancies rarely occurred with T/I dams or sires older than 5 months of age. These breeding problems were related to the combination of IL-10 and TNF deficiency, since similar problems were uncommon when single knockout *Il10* ^−/−^ or *Tnf* ^−/−^ mice were similarly maintained under Helicobacter-free conditions in the same facility. We routinely propagated the line using T-Het/I dams and T/I sires.

**Figure 1:**
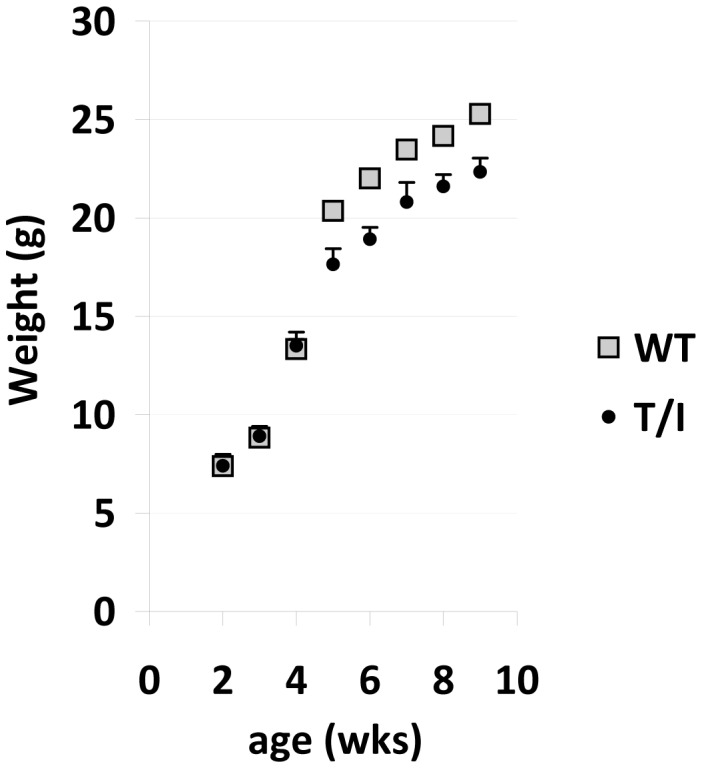
Body weights of T/I mice begin to lag soon after weaning. Mean ± SEM body weights are shown for wild type and T/I male mice between 2 and 9 wks of age. The number of litters and mice represented are 8 litters and 2–24 mice/point for wild type (WT) and 14 litters and 3–12 mice/point for T/I mice. Similar data was also obtained for the following strains that did not differ from wild type values at any of the 8 time points tested: *Il10* ^−/−^ (IL10 KO), 5 litters, 3–21 mice/point; *Tnf* ^−/−^ (TNF KO), 5 litters, 7–13 mice/point; T-het/I, 14 litters, 2–6 mice/point. The SEMs for WT mice ranged from 0.2 to 0.6 g and thus are hidden by the markers chosen. *indicates a significant difference between WT and T/I mice; p values ranged from 0.0001 to 0.03. Similar trends were seen in female mice (not shown).

When born to a healthy dam, T-het/I and T/I pups were of similar weight during the first 3 weeks of life and their weights were similar to those observed in age-matched wild type C57BL/6, *Il10* ^−/−^, or *Tnf* ^−/−^ pups raised in the same facility (Figure 1). However, the weights of T/I mice began to lag behind those of WT, T-het/I, *Il10* ^−/−^, and *Tnf* ^−/−^ mice soon after weaning (Figure 1 and data not shown). This led us to hypothesize that T/I mice might be susceptible to developing colitis spontaneously, in the absence of the specific triggering required to induce colitis in *Il10* ^−/−^ mice.

### TNF Deficiency Predisposes to Development of Spontaneous Colitis

**Figure 2:**
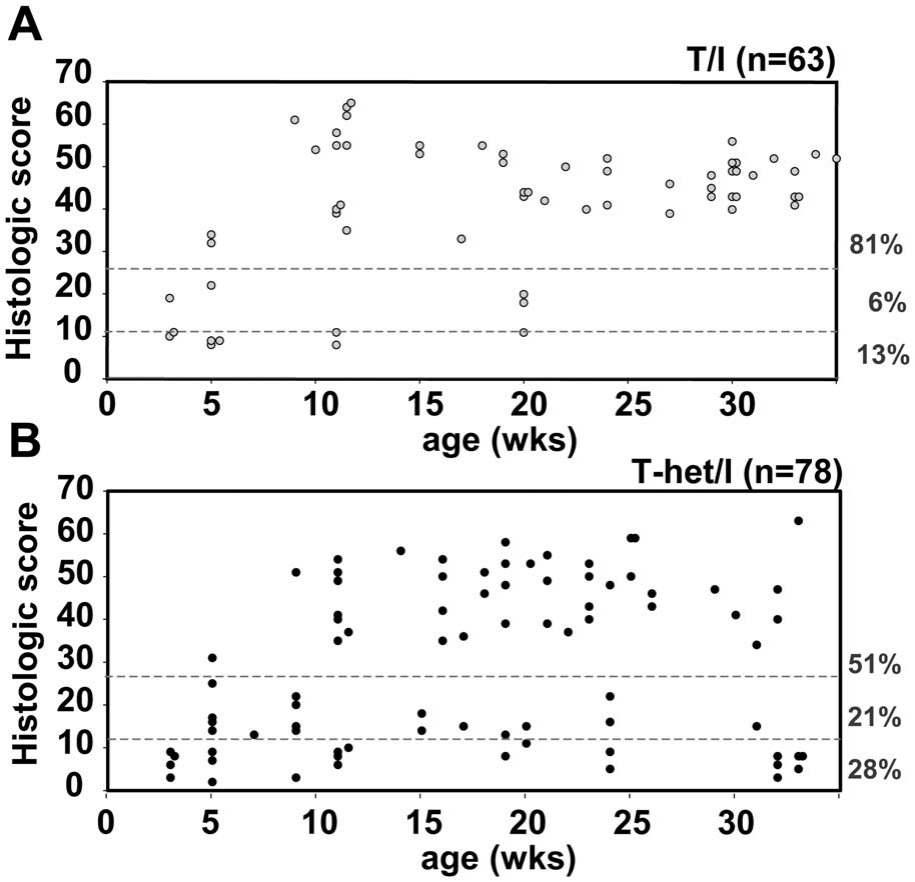
Spontaneous colitis in T/I and T-het/I mice. The colon histologic score is shown as a function of age for T/I (panel A) and T-het/I (panel B) mice. Each dot represents a single mouse studied. Dashed lines divide mice into groups with no colitis (histologic score ≤12), mild colitis (histologic scores between 13 and 24), and moderate to severe colitis (histologic score ≥25). The percentage of mice in each of these severity categories is shown at the right of each figure. Significantly fewer T-het/I mice had moderate to severe colitis compared with T/I mice (p = 0.0004; relative risk 0.39, 95% confidence interval 0.22–0.68).

WT and *Tnf* ^−/−^ mice housed in the same animal facility as T-het/I and T/I mice showed no evidence of spontaneous colitis (Table 1). Although some *Il10* ^−/−^ control mice examined at 8–12 wks of age had histologic scores consistent with mild colitis, the mean histologic score in this cohort did not differ significantly from what was observed in WT mice (Table 1). These results are consistent with our previous reports that *Il10* ^−/−^ mice on the C57BL/6 background are resistant to the development of spontaneous colitis when kept under clean helicobacter-free conditions [8], [9]. However, we found that T/I mice spontaneously developed colitis under these same clean conditions (Figure 2A). The incidence and severity of spontaneous colitis in T/I mice increased with age. Minimal to no colon inflammation was present at 3 wks of age, but colitis was evident in some mice by 5 weeks of age. Overall, colitis was scored as moderate to severe in 81% of T/I mice examined between the ages of 3 and 35 wks (n = 63). Furthermore, all T/I mice scored with no (13%) or mild colitis (6%) were less than 20 wks of age (Figure 2A; Table 1).

**Table 1:** Severity of Spontaneous Colitis as a Function of Age.^a^

	3 wk	5 wk	8–12 wk	14–25 wk	26+ wk b
WT	12±2 (n = 4)	7±2 (n = 4)	4±1 (n = 11)	N.D.	4±1 (n = 16)
*Tnf* ^−/−^	8±1 (n = 4)	6±1 (n = 5)	4±1 (n = 6)	N.D.	8±1 (n = 13)
*Il10* ^−/−^	2±1 (n = 4)	9±1 (n = 5)	19±4 (n = 10)	N.D.	11±1 (n = 23)
T-het/I	7±1 (n = 4)	15±3 (n = 8)	27±4 (n = 17)^c^	37±3 (n = 36)	27±5 (n = 16)c,d
T/I	13±3 (n = 3)	19±5 (n = 6)	46±5 (n = 14)^c^	42±3 (n = 18)	47±1 (n = 22)c,d

N.D.  =  Not done.

a Data shown is the histologic score mean ± SEM for each genotype and age group studied. A score of >12 indicates at least mild colitis.

b The ranges of ages studied were: WT = 29–44 wks; *Tnf* ^−/−^  = 30–42 wks; *Il10* ^−/−^  = 29–49 wks; T-het/I = 26–46 wks; T/I = 27–35 wks.

c p<0.001 vs WT; one way ANOVA, with Dunnett’s post-test.

d p<0.001 vs. each other; one way ANOVA, with Tukey’s post-test.

Some mice that were heterozygous for TNF deficiency (T-het/I mice) also developed colitis soon after weaning, but were significantly less likely to do so compared with T/I mice (relative risk 0.39, 95% confidence interval 0.22–0.68; p = 0.0004) (Figure 2B). Overall, colitis was scored as moderate to severe in only 51% of T-het/I mice studied (n = 78). In marked contrast to T/I mice where 100% of mice >30 wks of age had moderate to severe colitis (n = 17), only 38% of T-het/I mice examined at >30 wks of age (n = 13) had moderate to severe colitis (Figure 2B). Although the mean histologic scores for T-het/I mice were lower than T/I mice for the age ranges of 8–12 wks and 26+ wks (Table 1), this difference primarily reflected the decreased % of T-het/I mice with colitis examined at these ages rather than any genotype-related difference in colitis severity *per se* (Figure 2).

**Figure 3:**
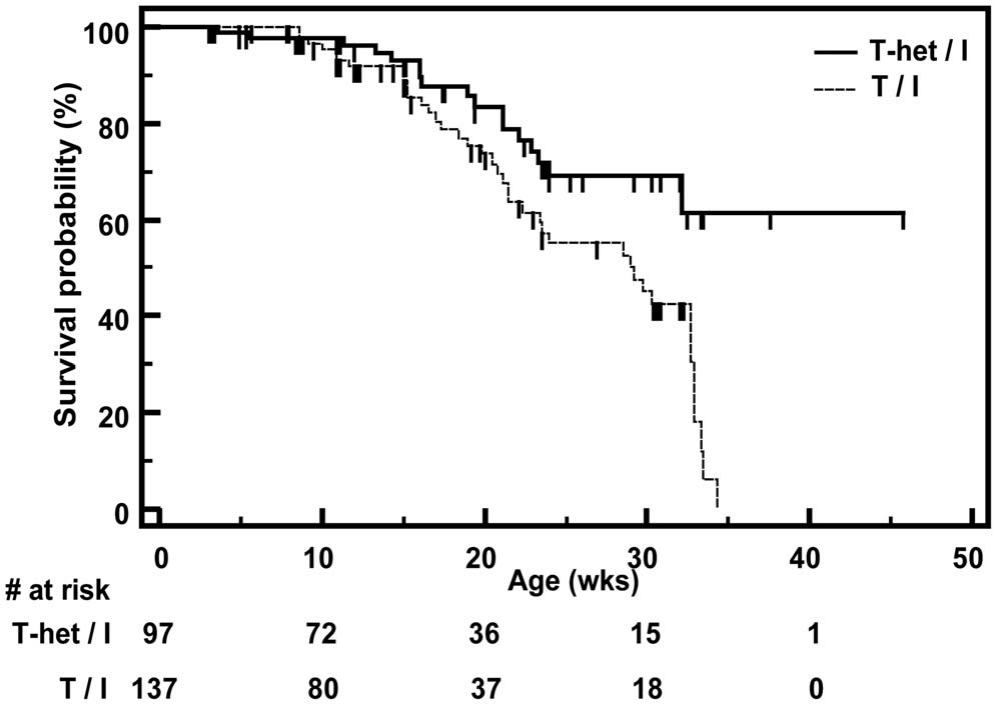
Kaplan-Meier survival curves for T/I and T-het/I mice. The probability of survival as a function of time is shown for T-het/I vs. T/I mice. Censored data for mice euthanized prior to achieving humane endpoints is marked with a small vertical line. The number of mice at risk is provided beneath the graph. T/I mice were significantly more likely to die or to require euthanasia for humane reasons than T-het/I mice (p = 0.0032; log rank test).

The histologic scores for T-het/I and T/I mice described above included mice that required euthanasia for colitis-related humane endpoints, in addition to mice that were prospectively euthanized for tissue harvest at pre-specified time points. Kaplan-Meier analysis showed that T/I mice were significantly more likely to die or to require euthanasia for humane reasons than T-het/I mice (hazard ratio  = 2.28; 95% confidence interval: 1.35–3.85; p = 0.0032; log rank test) (Figure 3). Taken together with the data on colitis severity (e.g. histologic scores), these data clearly show that TNF is not required for development of clinically significant severe colitis in *Il10* ^−/−^ mice. To the contrary, inability to produce sufficient TNF is a strong risk factor for the spontaneous development of severe and potentially fatal IBD in a setting of IL-10 deficiency.

### Response of T/I Mice to Intestinal Barrier Disruption Via NSAID Exposure

NSAIDs such as piroxicam enhance apoptosis of colonic epithelium, leading to defects in the mucosal barrier that allow bacteria within the intestinal lumen to contact and potentially activate immune cells located in the lamina propria [12]. To determine how defects in TNF production affected responses to mucosal barrier disruption, T-het/I and T/I mice were exposed to 200 ppm of the NSAID piroxicam in their diet for 7 days; piroxicam was discontinued and mice were observed for an additional 16 days before euthanasia for assessment of colitis severity. Although some T-het/I mice appeared resistant to developing colitis spontaneously (Figure 2B), piroxicam-exposed T-het/I mice uniformly developed colitis (mean histologic score  = 38±3; n = 14). 86% of the T-het/I mice survived to the planned study endpoint 16 days after discontinuation of piroxicam. The severity and histologic pattern of colon inflammation observed in piroxicam-exposed T-het/I mice was similar to that observed in T-het/I mice that developed colitis spontaneously and did not differ from what was observed in T/I mice similarly exposed to piroxicam (mean histologic score  = 45±3; n = 5; p = 0.11). Although the severity of any spontaneous colitis in these T/I mice could not be assessed prior to piroxicam exposure, their colitis severity post-piroxicam was similar to what was observed in T/I mice that developed spontaneous colitis in the absence of piroxicam exposure (compare with Table 1). However only 17% (1 of 6) of piroxicam-exposed T/I mice survived until the planned study endpoint 16 days after discontinuation of piroxicam (p = 0.007; Fisher’s exact test).

### Histology of Colitis in T/I Mice Closely Resembles that Seen in Human UC

**Figure 4:**
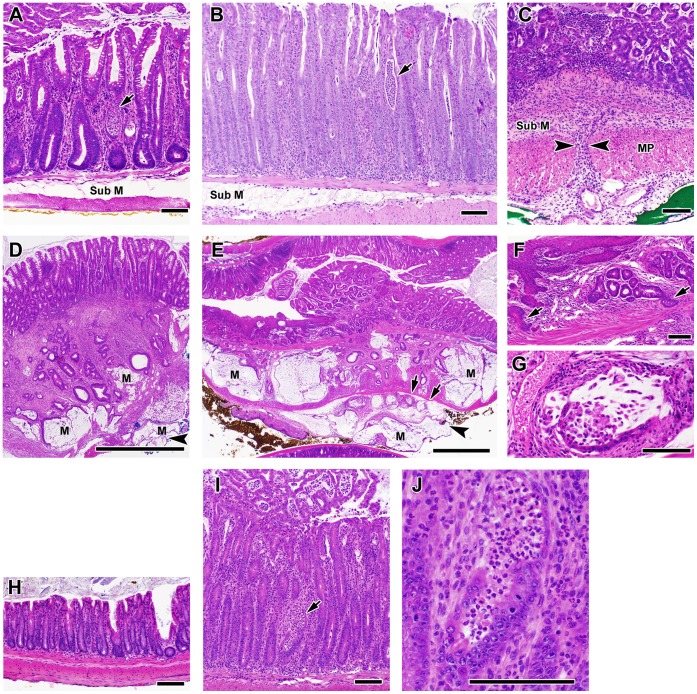
Colitis and inflammation-associated colon cancer in T/I and T-het/I mice. **A**. Spontaneous colitis in T/I mice (rectum shown) is characterized by epithelial hyperplasia and infiltration of the lamina propria with inflammatory cells, including large numbers of neutrophils. Crypt abcesses are common (arrow). Inflammation typically involves the mucosa only, with little to no inflammation present in the submucosa (Sub M). **B**. Piroxicam-triggered colitis (shown) and spontaneous colitis (not shown) in T-het/I mice has a similar histologic apprearance to colitis in T/I mice (rectum shown; arrow indicates crypt abcess). **C**. In contrast, colitis (rectum shown) in *Il10* ^−/−^ mice is typically transmural as indicated by arrowheads, with inflammation present in the submucosa and/or muscularis propria (MP). **D,** **E**. Colon cancers that develop in T/I mice (and T-het/I mice, not shown) are typically mucinous adenocarcinomas that may show extensive local invasion. The tumor shown in D is from proximal colon; the tumor in E is from rectum. M indicates mucin lakes. Ink on tumor (arrowheads) indicates invasion completely through the bowel wall (the arrows in E point to the serosal surface). **F**. T/I (and T-het/I, not shown) mice with colitis often exhibited squamous metaplasia in the rectum, that could give rise to carcinomas with both glandular and sqamous features (arrows). **G**. Higher magnification view of mucinous adenocarcinoma in a T/I mouse. **H,** **I**. T/I mice given placebo food spontaneously developed severe colitis by 8 wks of age (I), whereas T/I mice given matched food containing antibiotics showed no evidence of inflammation at this time point (H). Arrow indicates a crypt abcess, shown at higher magnification in J. Scale bar  = 100 µm in A - C, F - J and 1 mm in D and E.

**Figure 5:**
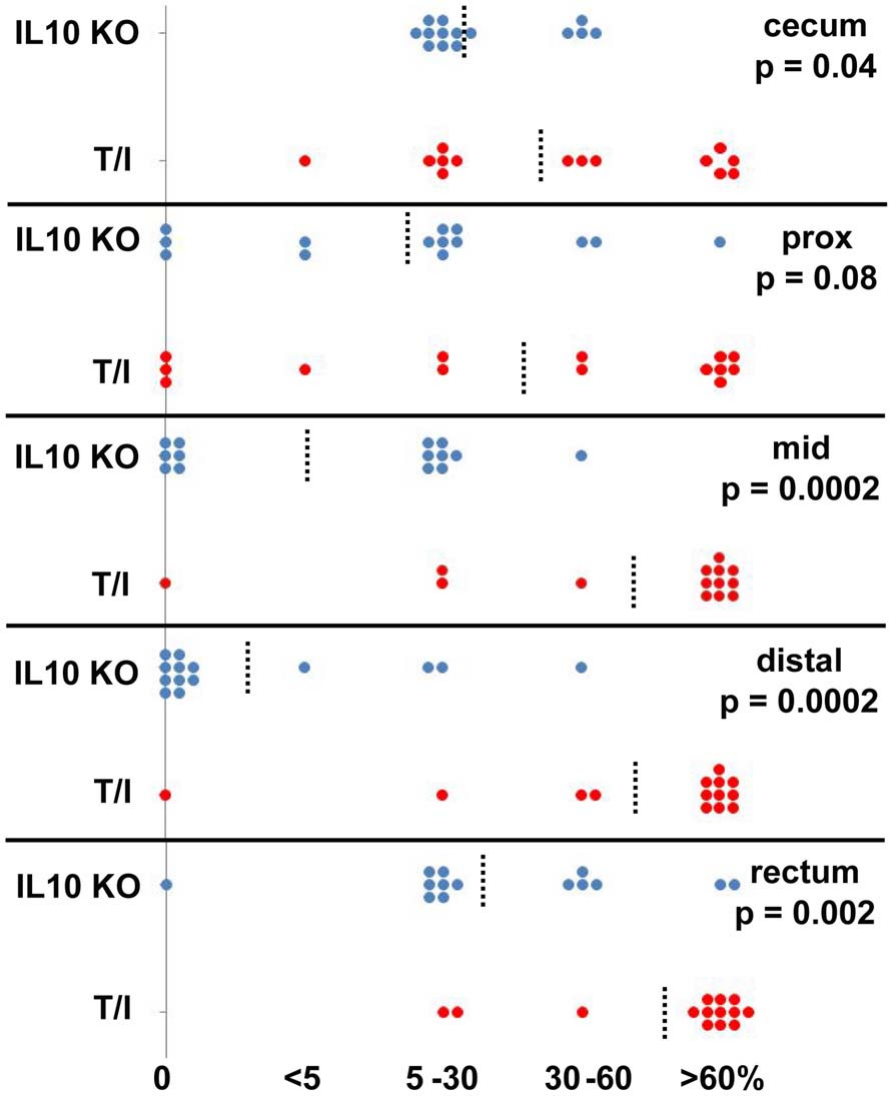
Distribution of colonic inflammation in *Il10* ^−/−^ (IL10 KO) vs. T/I mice. The % of colonic area that exhibited inflammatory changes in each segment of the colon (an assessment required for calculation of histologic scores [12]) is shown for a cohort of *Il10* ^−/−^ mice with colitis triggered by exposure to piroxicam (n = 14) and an age-matched cohort of 11–12 wk T/I mice with spontaneous colitis (n = 14). The dashed line indicates the median area involved in each colon segment. The p values shown represent comparison of disease extent (involving 0–60% vs. >60% of the segment area) in the 2 genotypes using Fisher’s exact test.

The terminal colon/rectum was inflamed in 100% of T/I mice (n = 40) examined histologically at ≥15 wks of age. Inflammation typically was continuous, beginning at the rectum and typically progressed proximally in a linear fashion to involve most or all of the colon. However in rare cases, T/I mice had inflammation limited to the terminal colon/rectum (n = 1, at 5 wks) or to the distal colon plus rectum (n = 2, at 5 wks). The lamina propria of the rectum and more proximal inflamed tissues of T/I mice was packed with inflammatory cells including large numbers of neutrophils. Crypt abcesses and ulceration were common, but inflammation generally did not extend past the muscularis mucosae and into the submucosa (Figure 4A). The pattern and severity of inflammation that was observed in T-het/I mice with spontaneous (not shown) or piroxicam-triggered colitis (Figure 4B) was similar to that observed in T/I mice (Figure 4A). In contrast, when triggered in mice deficient in IL-10 alone, colon inflammation was characteristically transmural (Figure 4C), with regions of inflammation widely separated by non-inflamed regions (“skip lesions”). A comparison of the location and extent of inflammation in *Il10* ^−/−^ vs. T/I mice is presented in Figure 5. Colitis in *Il10* ^−/−^ mice was typically most extensive in the proximal colon, followed by the cecum and rectum; the median area involved in each segment was ≤30%. In contrast, many T/I mice had the highest possible extent score, with inflammatory changes present in >60% of the area examined in each colon segment. T/I mice thus had a significantly larger extent of disease in 4 of the 5 colonic regions examined compared to *Il10* ^−/−^ mice (Figure 5). Based on these features, the histology of colitis in TNF-deficient T/I mice (and TNF-haplo-insufficient T-het/I mice) closely resembled what is typically seen in human UC, while colitis in mice singly deficient in IL-10 more closely resembled that seen in human CD.

### Inflammation-associated Colonic Neoplasia in T/I and T-het/I Mice

Colon inflammation has been shown to predispose to development of inflammation-associated neoplasia in both humans and in mouse models of IBD, including *Il10* ^−/−^ mice [8]–[10], [22]–[27]. We found the same to be true in T/I and T-het/I mice with colitis. 93% of the T/I mice (n = 40) examined at >15 wks of age had moderate to severe colitis. Neoplastic lesions were observed in the colons of 63% this cohort, with a mean of 2 neoplastic lesions/mouse (range 0–4). The majority of these lesions were invasive mucinous adenocarcinomas characterized by relatively bland-looking columnar epithelial cells and extensive production of mucin forming “mucin lakes” (Figure 4D, E, G). Progression to locally advanced disease in which tumor invaded through the wall of the colon (Figure 4D–G) was common in mice older than 25 wks, but no lymph node or other metastases were observed. Extensive squamous metaplasia was frequently present in the rectum of these mice and some carcinomas with a squamous component were observed, particularly in the rectum (Figure 4F). Only 65% of T-het/I mice examined at ≥15 wks of age had moderate to severe colitis (n = 52). The incidence of neoplasia in this cohort was 54%, with a mean of 2 neoplastic lesions/mouse (range 0–6; n = 36). All mice with neoplasia had histologic scores ≥40. Thus, similar to humans with UC [22]–[24], T/I and T-het/I mice have an enhanced risk of developing colon cancer in the setting of inflammation.

### Antibiotics Prevent Development of Spontaneous Colitis in T/I Mice

IBD is currently hypothesized to result from a pathogenic immune response against colitis-inducing (colitogenic) microbiota in a susceptible host. To test the hypothesis that colitis in T/I mice results from pathogenic immune responses to increases in colitogenic bacteria that naturally occur at the time of weaning [28], T/I mice were weaned to consume diets with or without a 4 drug cocktail of amoxicillin, clarithromycin, metronidazole, and omeprazole until 8 wks of age. Although this 4 drug cocktail was originally developed for eradication of helicobacter infections in mice, we have shown that it also suppresses or eradicates colitogenic bacteria and thus is also able to prevent the development of colitis in helicobacter-free *Il10* ^−/−^ mice that are exposed to piroxicam [8]. T/I mice that received food without antibiotics uniformly exhibited severe colitis when examined at 8 wks of age (mean histologic score ± SEM  = 49±4; n = 8; Figure 4I, J). In contrast, T/I mice that received this 4 drug antibiotic cocktail in their food from the time of weaning had no evidence of colon inflammation at 8 wks of age (mean histologic score ± SEM  = 2±1; n = 5; Figure 4H).

### Systemic Responses of T/I Mice to Bacterial Challenge

The above studies definitively show that spontaneous development of colitis in T/I mice is driven by colitogenic bacteria. The cytokine TNF is typically a major component of antibacterial responses and has been hypothesized to be critical for IBD pathogenesis, since therapy with certain anti-TNF drugs has been highly effective in treating IBD in humans. However, our studies show that the ability to produce TNF is not required for the development of IBD in *Il10* ^−/−^ mice. To begin to understand how TNF and IL-10 may influence responses to microbial exposure, we determined how mice with varying combinations of wild type and null alleles for *Tnf* and *Il10* responded systemically to *in vivo* challenge with bacterial lipopolysaccharide (LPS) or endogenous intestinal microbiota.

TNF levels were undetectable in the serum of unchallenged WT, *Il10* ^−/−^, *Tnf* ^−/−^, T-het/I, and T/I mice. As expected, levels of TNF present in serum obtained 90 min after exposure to LPS derived from *E. coli* were lower in mice with one null *Tnf* allele, with levels ∼20–30% of wild type (Table 2). However, mice with one or more null *Il10* alleles showed increased serum TNF responses to LPS, in a dose-dependent fashion. T-het/I mice thus had LPS-induced serum TNF levels that were ∼60% of those seen in wild type mice. *Il10* ^−/−^ mice with 2 wild type *Tnf* alleles had LPS-induced serum TNF levels 160% higher than those observed in wild type mice (Table 2).

**Table 2:** Serum TNF Responses to LPS Challenge.*

	WT (*Il10* ^+/+^)	Heterozygous(*Il10* ^+/−^)	Knock-Out (*Il10* ^−/−^)
WT (*Tnf* ^+/+^)	**9165**±1671 (n = 12)	Not Done	**17,586**±3206 (n = 9)^a^
Heterozygous(*Tnf* ^+/−^)	**1659**±145 (n = 5)	**2357**±323 (n = 7)	**5,417**±316 (n = 5)^b^
Knockout (*Tnf* ^−/−^)	0 (n = 5)	0 (n = 7)	0 (n = 5)

* Data shown are mean ± SEM, in pg/ml as measured by enzyme immunoassay. Mice heterozygous for IL-10 alone were not studied since they were not generated in large numbers by our chosen breeding scheme.

a p<0.05 vs. WT; ANOVA with Tukey’s post-test.

b p<0.05 vs. *Il10* ^−/−^; ANOVA with Tukey’s post-test.

Multiplex immunoassays were used to screen WT, *Il10* ^−/−^, *Tnf* ^−/−^, T-het/I, and T/I mice for differences in production of 15 additional cytokines (Table 3; Table S1). LPS challenge markedly increased serum levels of IL-4, IL-5, IL-7, IL-9, IL-13, and IL-15 above levels observed in the serum of non-challenged WT, *Il10* ^−/−^, *Tnf* ^−/−^, T-het/I, and T/I mice, but the levels of these cytokines did not differ between the genotypes (Table S1). LPS-stimulated levels of IFN-γ, IL-1α, IL-1β, IL-12p70, and IL-17 in the serum of *Il10* ^−/−^, *Tnf* ^−/−^, T-het/I, and T/I mice were similar to those measured in WT mice (Table 3). However, serum levels of IFN-γ, IL-1α, IL-6, and IL-17 were elevated at baseline in the serum of T/I mice (these mice had colitis at the time of assessment) compared with WT mice and IFN-γ and IL-17 did not significantly increase in the serum of T/I mice following challenge with LPS (Table 3). LPS-challenged T/I mice also had elevated serum levels of IL-12/IL-23 p40 and the T cell activating cytokine IL-2 compared to WT mice (Table 3). Like T/I mice, unmanipulated T-het/I mice also had elevated baseline serum levels of IFN-γ and IL-6 compared to WT mice (Table 3). However, contrary to what was observed in T/I mice, serum levels of both IFN-γ and IL-6 were markedly and significantly increased when T-het/I mice were LPS-challenged. Serum IL-6 greatly increased in *Tnf* ^−/−^ mice challenged with LPS, but the numerical increase was significantly less than was observed in LPS-challenged WT mice (Table 3). Since serum samples were obtained 90 min after LPS stimulation, these changes in serum cytokine levels relative to unmanipulated mice represent either innate immune responses or activation of *in vivo* primed T cells. The marked elevation of IL-12/IL-23 p40 observed in LPS-challenged T/I mice without a corresponding increase in levels of IL-12 p70 suggests elevation of the Th17-inducing cytokine IL-23 in these mice, rather than IL-12 *per se*.

**Table 3:** Systemic Cytokine Responses to Bacterial Challenge.

Unmanipulated^a^						LPS-challenged (90 min)^b^					Piroxicam (42 hr)^c^				
	WT	IL10 KO	TNF KO	T-het/I	T/I	WT	IL10 KO	TNF KO	T-het/I	T/I	WT	IL10 KO	TNF KO	T-het/I	T/I
**IFN-γ**	4±2	6±3	11±5	**30±19** *	**198±118** *	167±8	154±15	113±12	153±8	145±35	13±7	17±4	2±2	34±12	45±18
**IL-1α**	270±70	210±34	213±22	235±50	**707±56** *	2162±107	2056±103	1882±115	2105±110	2068±277	531±47	618±90	578±108	1034±357	1012±415
**IL-1β**	24±11	18±12	12±8	18±12	86±18	275±21	340±66	251±19	355±20	588±192	38±7	79±9	19±7	105±31	**160±53** *
**IL-2**	7±1	6±1	7±1	13±4	11±3	24±1	27±2	20±1	23±1	**40±6** *	10±2	16±1	7±1	15±1	**71±53** *
**IL-6**	12±2	5±2	7±1	**60±36** *	**98±13** *	75330±2131	72731±3261	**56175±4123** *	84233±2447	81514±3943	47±5	98±16	97±55	2887±2768	3772±3519
**IL-10**	0	0	0	0	0	4536±687	0	3105±654	0	0	0	0	0	0	0
**IL-12/ IL-23 p40**	37±4	41±3	50±4	28±12	48±16	211±20	285±42	186±11	288±64	**1991±906** *	33±9	34±5	18±4	28±3	87±62
**IL-12 p70**	63±28	57±25	92±28	55±27	66±43	365±15	400±58	340±26	356±32	401±146	52±34	16±16	17±12	36±26	433±433
**IL-17**	16±1	13±3	18±5	51±23	**128±44** *	142±3	124±11	109±5	125±7	263±144	19±4	34±2	16±3	66±22	**550±495** *

* indicates p<0.05, compared with wild type (ANOVA with Dunnett’s post-test).

a Serum was obtained from unmanipulated mice of the indicated genotypes at 11–12 wks of age. At this time point, all of the T/I and T-het/I mice studied had at least mild colitis, whereas WT, *Il10* ^−/−^, and *Tnf* ^−/−^ mice had no evidence of colon inflammation. Data shown is the mean ± SEM of 5 mice tested per group.

b Serum was obtained 90 min after i.p. injection of 100 µg LPS. Data shown is the mean ± SEM of 5 mice tested per group.

c Serum was obtained 42 hrs after initiating a diet containing 200 ppm of the NSAID piroxicam. Data shown is the mean ± SEM of 4 mice tested per group.

To assess whether similar patterns of cytokine responses occur when mice of these genotypes are exposed to intestinal microbiota and their products *in vivo,* serum cytokines were measured in WT, *Il10* ^−/−^, *Tnf* ^−/−^, T-het/I, and T/I mice given the NSAID piroxicam in their food for 42 hrs. We previously showed that piroxicam degraded the intestinal barrier and enhanced exposure to intestinal microbiota [12]. For most of the cytokines measured, piroxicam exposure generated similar levels of cytokines in the serum of WT, *Il10* ^−/−^, *Tnf* ^−/−^, T-het/I, and T/I mice (Table 3; Table S1). However, piroxicam-exposed T/I mice showed increased serum levels of the general immune activator IL-1β, the T cell stimulatory cytokine IL-2, and the Th17 cytokine IL-17 (p<0.05) compared to piroxicam-exposed WT mice. A strong trend toward increased serum levels of the Th2/Th17 cytokine IL-6 was also observed in both T-het/I and T/I mice, albeit with wide variability in degree of elevation between individuals (Table 3). Such variability may be physiologic, since animals may voluntarily ingest the piroxicam at different times after its introduction (in the diet) and levels of pro-inflammatory cytokines are known to vary markedly with time, particularly early in the inflammatory process. Overall, the patterns of cytokine secretion observed following *in vivo* challenge with LPS or NSAID suggested general immune activation, with polarization toward the Th17 pathway, a response that typically occurs in response to bacterial exposure.

## Discussion

The impressive clinical success of anti-TNF therapy for treating humans with IBD has led to the hypothesis that TNF is a major regulator of inflammation in IBD. However, the work presented here clearly demonstrates that TNF is not required for the development of IBD, at least in *Il10* ^−/−^ mice, a model with genetic, clinical, and histologic features that closely resemble human Crohn disease (CD). Instead, mice with either no TNF (T/I) or reduced ability to produce TNF (T-het/I) spontaneously developed colitis under clean, helicobacter-free conditions. The clinical and histologic pattern of inflammation in T/I and T-het/I mice closely resembled that observed in human ulcerative colitis (UC) and, like humans with UC, T/I and T-het/I mice were at high risk of developing inflammation-associated colon cancers. Decreased or absent TNF production alone was not sufficient to result in colitis. Absence of both TNF and IL-10 production resulted in a Th17-polarized pattern of cytokine production in response to systemic bacterial challenge. Spontaneous development of colitis in T/I mice could be prevented by administration of antibiotics prior to and after weaning, directly implicating the exposure to colitogenic bacteria that occurs around the time of weaning in disease pathogenesis.

IBD has been hypothesized to result from an aberrant immune response to enteric bacteria that occurs in a genetically susceptible host. Based on data obtained from both animal models and humans, we have proposed that the development of IBD requires 3 factors [29]. First, bacterial antigens and adjuvants capable of triggering colitis must be present within the intestine. Second, the mucosal barrier must be compromised so that the bacterial antigens and adjuvants present within the intestinal lumen can come in contact with the innate and adaptive immune cells to generate responses. And third, the host must have a defect in immune regulation that allows persistence of immune responses against these antigens. This three-factor model can potentially explain how the known susceptibility alleles and IBD-related triggers in existing murine models result in the development of chronic colitis [29]. Our work and that of others suggests that bacterial antigens and adjuvants capable of causing colitis are present in all animals that are not raised under germ-free conditions. Deficiency of IL-10 results in defective immune regulation. The model therefore predicts that the function of the mucosal barrier will determine whether or not colitis develops in in specific sets of *Il10* ^−/−^ animals.

Exposure to piroxicam is known to compromise the intestinal mucosal barrier. That the severity of inflammation in T/I mice was not increased with piroxicam exposure provides additional evidence that the mucosal barrier is already defective in these mice due to absence of TNF. Our studies were unable to separate the effects of TNF deficiency on the mucosal barrier of T/I mice from those of inflammation, due to the early spontaneous development of colitis in T/I mice. However, stochastic factors that affect mucosal barrier integrity may determine the presence or absence of spontaneous colitis in any given T-het/I mouse, since T-het/I mice were uniformly susceptible to development of severe colitis when their mucosal barrier was definitively disrupted by exposure to piroxicam. The increased mortality seen in piroxicam-exposed T/I vs. T-het/I mice further confirms the importance of TNF as a protective factor in the setting of exposure to bacteria and bacterial products.

In retrospect, the generally poor reproductive performance of T/I dams was almost certainly due to the near universal early development of severe IBD that either affected mating behavior, fertility, or the ability of dams to adequately nourish their pups. Effects of colon inflammation on reproductive performance have also been documented in humans (reviewed in [30]), although both fertility and pregnancy outcomes can be good if disease is well-controlled. Spontaneous development of IBD in T/I mice was accompanied by a drop-off in the trajectory of weight gain soon after weaning, without other obvious behavioral or physical signs of illness. That the ∼60% of wild-type levels of TNF made by T-het/I mice was sufficient to protect about half the mice from developing IBD spontaneously suggests that a threshold level of TNF production is protective against IBD, at least in the setting of IL-10 deficiency. That enhanced TNF production (e.g. 160% of wild type levels, as observed in *Il10* ^−/−^ mice) cannot prevent the development of IBD under conditions of massive mucosal barrier compromise (e.g. NSAID exposure or helicobacter infection) further suggests that the roles of TNF in barrier maintenance vs. inflammation are potentially separable.

In this study, striking UC-like vs. CD-like phenotypic differences were observed in the distribution and histologic characteristics of colitis of *Il10* ^−/−^ mice based on changes in a single gene that affected TNF production. Mutations in the IL-10 receptor have been documented to predispose to CD in humans [1]. However, a large genome-wide association study in UC patients identified association with a SNP immediately flanking the IL10 gene, suggesting that defective IL10 function was central to the pathogenesis of the UC subtype of human IBD [3]. Genes encoding multiple components in the Th17 pathway (*IL23R*, *IL12B*, *JAK2* and *STAT3*) have previously been found to be associated with both UC and CD in humans (reviewed in [31]). Genes involved in maintaining the integrity of the intestinal barrier have also been strongly associated with the pathogenesis of UC [31]). Our animal model provides a mechanistic basis that unifies these seemingly disparate observations, by identifying bacterially-induced TNF production as a mechanism that governs the overall pattern of inflammatory responses as well as mucosal barrier function. While humans rarely have deletion of the TNF gene, relative TNF insufficiency (as seen in T-het/I mice) due to variations in other regulatory genes could either compromise the mucosal barrier directly and/or drive IBD development as UC (as opposed to CD) when the mucosal barrier is compromised via other mechanisms.

Humans with UC have a markedly increased risk of developing colon cancer compared to the general population [24]. Our data show that T/I and T-het/I mice with UC-like IBD also have a high risk of IBD-associated colon cancer. The geographically continuous inflammation observed in both humans with UC and in T/I and T-het/I mice would be expected to maximize inflammation-associated mutagenesis, since all epithelial cells in the colon would potentially be exposed to mutagenesis resulting from inflammatory mediators. Colon carcinogenesis can clearly progress quite rapidly in T/I mice with colitis, since one or more colonic neoplastic lesions were observed in two-thirds of T/I mice examined at ≥15 wks of age. The rapidity of cancer development and progression makes the T/I model ideal for study of mechanisms of inflammation-associated carcinogenesis and for testing candidate chemopreventative therapies. Of note, similarly rapid kinetics of cancer development were previously reported in *Helicobacter bilis*-infected *Smad3* ^−/−^ mice, where approximately two-thirds develop inflammation-associated mucinous colon carcinomas as early as 6 weeks post-infection [32].

Exposure to potential microbial pathogens stimulates activation of innate immune cells and triggers antigen-presenting cells to present microbial antigens to naive T cells. Subsequent T cell differentiation to Th1, Th2, or Th17 cells depends on the cytokine milieu produced by these early responders. Th17 differentiation is favored by high levels of IL-6, IL-23, and TGF-β. IL-12 and IFN-γ favor Th1 responses, while IL-4 favors Th2 responses [33]. Before the recent discovery of Th17 cells, human CD was thought to be due to Th1 activation, while UC was thought to result from Th2 activation. Subsequent data has called into question the involvement of Th2 cells in UC and shown the importance of Th17 activation in both forms of IBD. While some cytokines are produced primarily by innate immune cells or by adaptive immune cells, others are commonly produced by both types of cells. The serum obtained 90 min after LPS challenge primarily reflects innate immune responses. Serum cytokine levels in unmanipulated mice reflect ongoing immune activation that would include both innate and adaptive immune responses. Serum cytokine levels 42 hrs after piroxicam challenge also would likely be due to both innate and adaptive immune responses, particularly in mice that had been primed *in vivo*. T/I mice clearly differ from the other genotypes tested in regard to their increased production of the Th17-type cytokines IL-6 and IL-17 in the absence of manipulation and increased IL-17 production when challenged by piroxicam. T/I mice also have significantly increased production of the T cell activator IL-2 and the Th17-inducing IL12/IL23 p40 when stimulated by LPS. The increased IFN-γ and IL-1α that was also observed in the serum of unmanipulated T/I mice may reflect that these mice have (spontaneous) colitis while the other genotypes of mice studied do not, but additional studies will be required to determine the cellular source(s) of these cytokines. Interestingly, although the source of the elevated serum IL-1β in T/I mice challenged with piroxicam is also not known, enhanced IL-1β production has been shown to enhance Th17-mediated inflammation, particularly when produced by dendritic cells [6]. Taken together, our data thus support the hypothesis that the UC-like IBD in T-het/I and T/I mice is driven primarily by Th17-mediated inflammation in response to exposure to colitogenic bacteria via a leaky mucosal barrier. Additional studies of local colonic cytokine production and infiltrating cell populations will be require to fully test this hypothesis, since although systemic cytokine levels provide a powerful method to address how T/I and T-het/I mice respond to bacterial challenge, they are of limited use for assessing localized intestinal inflammation.

TNF is well-known to be required for protection against bacterial infections. Indeed, genetic absence or pharmacological attenuation of the TNF response either systemically or in certain “early responder” immune cell types (e.g. mast cells) has been shown to predispose to increased morbidity and mortality from infections in both mice and humans [34]–[36]. Delayed or ineffective clearance of bacteria that penetrate the intestinal mucosal barrier due to insufficient TNF production could prolong innate immune responses and enhance presentation of bacterial antigens to facilitate development of Th17-type adaptive immune responses. Our results suggest that IBD may not be so much due to an aberrant immune response against intestinal bacteria as to an (at least initially) appropriate immune response that may be slow or ineffective at eradicating invading bacteria. These important concepts have obvious implications for development of targeted therapies to either prevent initial development of IBD in susceptible individuals or specifically target differential mechanisms that may operate early and late in disease pathogenesis.

## Supporting Information

Table S1 *indicates p<0.05, compared with wild type (ANOVA with Dunnett’s post-test). **^a^** Serum was obtained from unmanipulated mice of the indicated genotypes at 11–12 wks of age. At this time point, all of the T/I and T-het/I mice studied had at least mild colitis, whereas WT, *Il10* ^−/−^, and *Tnf* ^−/−^ mice had no evidence of colon inflammation. Data shown is the mean ± SEM of 5 mice tested per group. **^b^** Serum was obtained 90 min after i.p. injection of 100 µg LPS. Data shown is the mean ± SEM of 5 mice tested per group. **^c^** Serum was obtained 42 hrs after initiating a diet containing 200 ppm of the NSAID piroxicam. Data shown is the mean ± SEM of 4 mice tested per group.
(DOC)

## References

[1] Glocker E-O, Kotlarz D, Boztug K, Gertz EM, Schaffer AA, et al (2009) Inflammatory bowel disease and mutations affecting the interleukin-10 receptor. New Engl J Med. 361: 2033–2045. 10.1056/NEJMoa0907206 PMC2787406 19890111

[2] Van der Linde K, Boor PPC, Sandkuijl LA, Meijssen MAC, Savelkoul HFJ, et al (2003) A Gly15Arg mutation in the interleukin-10 gene reduces secretion of interleukin-10 in Crohn disease. Scand J Gastroenterol 38: 611–617. 12825869

[3] Franke A, Balschun T, Karlsen TH, Sventoraityte J, Nikolaus S, et al (2008) Sequence variants in IL10, ARPC2 and multiple other loci contribute to ulcerative colitis susceptibility. Nature Genet. 40: 1319–1323. 10.1038/ng.221 18836448

[4] Mottet C, Uhlig HH, Powrie F (2003) Cutting edge: Cure of colitis by CD4^+^CD25^+^ regulatory T cells. J Immunol. 170: 3939–3943. 10.4049/jimmunol.170.8.3939 12682220

[5] Murai M, Turovskaya O, Kim G, Madan R, Karp CL, et al (2009) Interleukin 10 acts on regulatory T cells to maintain expression of the transcription factor Foxp3 and suppressive function in mice with colitis. Nature Immunol. 10: 1178–1184. 10.1038/ni.1791 PMC2898179 19783988

[6] Wilke CM, Wang L, Wei S, Kryczek I, Huang E, et al (2011) Endogenous interleukin-10 constrains Th17 cells in patients with inflammatory bowel disease. J Translat Med. 9: 217. 10.1186/1479-5876-9-217 PMC3264534 22176654

[7] Kuhn R, Lohler J, Rennick D, Rajewsky K, Muller W (1993) Interleukin-10-deficient mice develop chronic enterocolitis. Cell 75: 263–274. 8402911 10.1016/0092-8674(93)80068-p

[8] Chichlowski M, Sharp JM, Vanderford DA, Myles MH, Hale LP (2008) *Helicobacter typhlonius* and *H. rodentium* differentially affect the severity of colon inflammation and inflammation-associated neoplasia in IL-10-deficient mice. Comp Med. 58: 534–541. PMC2710754 19149410

[9] Chichlowski M, Westwood GS, Abraham SN, Hale LP (2010) Role of mast cells in inflammatory bowel disease and inflammation-associated colorectal neoplasia. PLOS One 5: e12220. 20808919 10.1371/journal.pone.0012220 PMC2923184

[10] Hale LP, Perrera D, Gottfried MR, Maggio-Price L, Srinivasan S, et al (2007) Neonatal infection with *Helicobacter* species markedly accelerates the development of inflammation-associated colonic neoplasia in IL-10^−/−^ mice. Helicobacter. 12: 598–604. 10.1111/j.1523-5378.2007.00552.x 18001399

[11] Berg DJ, Zhang J, Weinstock JV, Ismail HF, Earle KA, et al (2002) Rapid development of colitis in NSAID-treated IL-10-deficient mice. Gastroenterol. 123: 1527–1542. 10.1053/gast.2002.1231527 12404228

[12] Hale LP, Gottfried MR, Swidsinski A (2005) Piroxicam treatment of IL-10 deficient mice enhances colon epithelial apoptosis and mucosal exposure to intestinal bacteria. Inflamm. Bowel Dis. 11: 1060–1069. 10.1097/01.mib.0000187582.90423.bc 16306768

[13] Present DH, Rutgeerts P, Targan S, Hanauer SB, Mayer L, et al (1999) Infliximab for the treatment of fistulas in patients with Crohn’s disease. N Engl J Med. 340: 1398–1405. 10.1056/NEJM199905063401804 10228190

[14] Nahar IK, Shojania K, Marra CA, Alamgir AH, Anis AH (2003) Infliximab treatment of rheumatoid arthritis and Crohn’s disease. Ann Pharmacol. 37: 1256–1265. 10.1345/aph.1C039 12921510

[15] Hanauer SB, Feagan BG, Lichtenstein GR, Mayer LF, Schreiber S, et al (2002) Maintenance infliximab for Crohn’s disease: the ACCENT I randomised trial. Lancet 359: 1541–1549. 12047962 10.1016/S0140-6736(02)08512-4

[16] Sands BE, Anderson FH, Bernstein CN, Chey WY, Feagan BG, et al (2004) Infliximab maintenance therapy for fistulizing Crohn’s disease. New Engl J Med. 350: 876–885. 10.1056/NEJMoa030815 14985485

[17] Rutgeerts P, Sandborn WJ, Feagan BG, Reinisch W, Olson A, et al (2005) Infliximab for induction and maintenance therapy for ulcerative colitis. New Engl J Med. 353: 2462–2476. 10.1056/NEJMoa050516 16339095

[18] Sandborn WJ, Hanauer SB, Katz S, Safdi M, Wolf DG, et al (2001) Etanercept for active Crohn’s disease: a randomized, double-blind, placebo-controlled trial. Gastroenterol. 121: 1088–1094. 10.1053/gast.2001.28674 11677200

[19] Hale LP, Cianciolo G (2008) Treatment of experimental colitis in mice with LMP-420, an inhibitor of TNF transcription. J. Inflamm. 5: 4. 10.1186/1476-9255-5-4 PMC2322983 18331642

[20] Burich A, Hershberg R, Waggie K, Zeng W, Brabb T, et al (2001) Helicobacter-induced inflammatory bowel disease in IL-10 and T cell-deficient mice. Am J Physiol Gastrointest Liver Physiol. 281: G764–G778. 10.1152/ajpgi.2001.281.3.G764 11518689

[21] Boivin GP, Washington K, Yang K, Ward JM, Pretlow TP, et al (2003) Pathology of mouse models of intestinal cancer: Consensus report and recommendations. Gastroenterol. 124: 762–777. 10.1053/gast.2003.50094 12612914

[22] Ekbom A, Helmick C, Zack M, Adami HO (1990) Ulcerative colitis and colorectal cancer. A population-based study. New Engl J Med. 323: 1228–1233. 10.1056/NEJM199011013231802 2215606

[23] Nordenholtz KE, Stowe SP, Stormont JM, Stowe MM, Chessin LN, et al (1995) The cause of death in inflammatory bowel disease: a comparison of death certificates and hospital charts in Rochester, New York. Am J Gastroenterol. 90: 927–932. 7771423

[24] Eaden JA, Abrams KR, Mayberry JF (2001) The risk of colorectal cancer in ulcerative colitis: a meta-analysis. Gut 48: 526–535. 11247898 10.1136/gut.48.4.526 PMC1728259

[25] Munkholm P (2003) Review article: the incidence and prevalence of colorectal cancer in inflammatory bowel disease. Aliment Pharmacol Ther 18: 1–5. 10.1046/j.1365-2036.18.s2.2.x 12950413

[26] Berg DJ, Davidson N, Kuhn R, Muller W, Menon S, et al (1996) Enterocolitis and colon cancer in interleukin-10-deficient mice are associated with aberrant cytokine production and CD4^+^ Th1-like responses. J Clin Invest. 98: 1010–1020. 10.1172/JCI118861 PMC507517 8770874

[27] Sturlan S, Oberhuber G, Beinhauer BG, Tichy B, Kappel S, et al (2001) Interleukin-10-deficient mice and inflammatory bowel disease associated cancer development. Carcinogenesis 22: 665–671. 11285204 10.1093/carcin/22.4.665

[28] Hill DA, Artis D (2010) Intestinal bacteria and regulation of immune cell homeostasis. Ann Rev Immunol. 28: 623–667. 10.1146/annurev-immunol-030409-101330 PMC5610356 20192812

[29] Chichlowski M, Hale LP (2008) Bacterial-mucosal interactions in inflammatory bowel disease – an alliance gone bad. Amer. J. Physiol. 295: G1139–G1149. 10.1152/ajpgi.90516.2008 PMC2604805 18927210

[30] Mahadevan U (2009) Pregnancy and inflammatory bowel disease. Gastroenterol Clin N Am. 38: 629–649. 10.1016/j.gtc.2009.07.006 19913206

[31] UK IBD Genetics Consortium & the Wellcome Trust Case Control Consortium (2009) Genome-wide association study of ulcerative colitis identifies three new susceptibility loci, including the *HNF4A* region. Nature Genet. 41: 1330–1334. 10.1038/ng.483 PMC2812019 19915572

[32] Ericsson AC, Myles M, Davis W, Ma L, Lewis M, et al (2010) Noninvasive detection of inflammation-associated colon cancer in a mouse model. Neoplasia 12: 1054–1065. 21170269 10.1593/neo.10940 PMC3003140

[33] Tang C, Chen S, Qian H, Huang W (2011) Interleukin-23: as a drug target for autoimmune inflammatory diseases. Immunol 135: 112–124. 10.1111/j.1365-2567.2011.03522.x PMC3277713 22044352

[34] Malaviya R, Ikeda T, Ross E, Abraham SN (1996) Mast cell modulation of neutrophil influx and bacterial clearance at sites of infection through TNF-α. Nature 381: 77–80. 8609993 10.1038/381077a0

[35] Echtenacher B, Mannel DN, Hultner L (1996) Critical protective role of mast cells in a model of acute septic peritonitis. Nature 103: 75–77. 10.1038/381075a0 8609992

[36] Stallmach A, Hagel S, Bruns T (2010) Adverse effects of biologics used for treating IBD. Best Pract & Res Clin Gastroenterol. 24: 167–182. 10.1016/j.bpg.2010.01.002 20227030

