# Supplementary material for: A Novel Murine Model of Inflammatory Bowel Disease and Inflammation-Associated Colon Cancer with Ulcerative Colitis-Like Features
Source: PLoS One. 2012 Jul 27;7(7):e41797. doi: 10.1371/journal.pone.0041797 (PMC3407062; doi:10.1371/journal.pone.0041797)
Supplement: Table S1 — *indicates p<0.05, compared with wild type (ANOVA with Dunnett’s post-test). a Serum was obtained from unmanipulated mice of the indicated genotypes at 11–12 wks of age. At this time point, all of the T/I and T-het/I mice studied had at least mild colitis, whereas WT, Il10 −/−, and Tnf −/− mice had no evidence of colon inflammation. Data shown is the mean ± SEM of 5 mice tested per group. b Serum was obtained 90 min after i.p. injection of 100 µg LPS. Data shown is the mean ± SEM of 5 mice tested per group. c Serum was obtained 42 hrs after initiating a diet containing 200 ppm of the NSAID piroxicam. Data shown is the mean ± SEM of 4 mice tested per group. (DOC) [file pone.0041797.s001.doc]

**Table S1: Additional Systemic Cytokine Responses to Bacterial Challenge**

Unmanipulated^a^ LPS-challenged (90 min)^b^ Piroxicam (42 hr)^c^

|  | **WT** | **IL10 KO** | **TNF KO** | **T-het/I** | **T/I** | **WT** | **IL10 KO** | **TNF KO** | **T-het/I** | **T/I** | **WT** | **IL10 KO** | **TNF KO** | **T-het/I** | **T/I** |
| --- | --- | --- | --- | --- | --- | --- | --- | --- | --- | --- | --- | --- | --- | --- | --- |
| **IL-4** | 4 ± 2 | 2 ± 1 | 2 ± 1 | 2 ± 1 | 7 ± 4 | 9 | 9 ± 2 | 6 ± 1 | 8 ± 1 | 21 ± 8 | 1 ± 1 | 0 | 0 | 1 ± 1 | 3 ± 3 |
| **IL-5** | 33 ± 9 | 17 ± 4 | 27 ± 5 | 13 ± 6 | 40 ± 16 | 122 ± 9 | 117 ± 10 | 127 ± 9 | 108 ± 5 | 118 ± 26 | 52 ± 8 | 24 ± 7 | 51 ± 9 | 53 ± 12 | 54 ± 24 |
| **IL-7** | 39 ± 25 | 25 ± 18 | 43 ± 11 | 17 ± 4 | 73 ± 40 | 87 ± 5 | 88 ± 7 | 117 ± 18 | 105 ± 13 | 107 ± 19 | 18 ± 7 | 27 ± 6 | 35 ± 11 | 29 ± 13 | 56 ± 30 |
| **IL-9** | 294± 114 | 255 ± 73 | 190 ± 50 | 324 ± 60 | 349 ± 83 | 1079 ± 111 | 1361 ± 224 | 953 ± 55 | 1065 ± 75 | 1892 ± 610 | 652 ± 275 | 693 ± 144 | 371 ± 68 | 1406 ± 500 | 309 ± 173 |
| **IL-13** | 51 ± 6 | 53 ± 11 | 65 ± 7 | 100 ± 34 | 55 ± 9 | 525 ± 25 | 523 ± 108 | 364 ± 27 | 444 ± 59 | 583 ± 101 | 73 ± 14 | 101 ± 8 | 45 ± 7 | 125 ± 26 | 147 ± 65 |
| **IL-15** | 32 ± 12 | 83 ± 49 | 132 ± 21 | 37 ± 14 | 173 ± 102 | 444 ± 54 | 437 ± 70 | 416 ± 24 | 412 ± 24 | 573 ± 120 | 49 ± 29 | 36 ± 13 | 81 ± 55 | 55 ± 43 | 105 ± 77 |
